# Chemical Composition, Antimicrobial, Antibiofilm Activities, and Cytotoxicity of the Essential Oil of *Dracocephalum botryoides* Stev.

**DOI:** 10.3390/plants15091416

**Published:** 2026-05-06

**Authors:** Gunay Jafarova, Kübra Erkan Türkmen, Javanshir Isayev, Ilkay Erdogan Orhan, Hikmet Katircioglu, Temel Ozek, Ebru Erdal

**Affiliations:** 1 Department of Pharmacognosy, Faculty of Pharmacy, Azerbaijan Medical University, AZ1022 Baku, Azerbaijan; isayev.cavanshir@amu.edu.az; 2 Department of Biology, Kamil Ozdag Faculty of Science, Karamanoglu Mehmetbey University, 70100 Karaman, Türkiye; kerkan@kmu.edu.tr; 3 Department of Pharmacognosy, Faculty of Pharmacy, Gazi University, 06330 Ankara, Türkiye; ilkayerdoganorhan@gmail.com; 4 Department of Pharmacognosy, Faculty of Pharmacy, Lokman Hekim University, 06510 Ankara, Türkiye; 5 Department of Biology Education, Faculty of Education, Gazi University, 06500 Ankara, Türkiye; hturk@gazi.edu.tr; 6 Department of Pharmacognosy, Faculty of Pharmacy, Yunus Emre Campus, Anadolu University, 26470 Eskisehir, Türkiye; tozek@anadolu.edu.tr; 7 Application and Research Center, Central Research Laboratory, Ankara Yıldırım Beyazıt University, 06800 Ankara, Türkiye; ebru.erdal.bio@gmail.com

**Keywords:** ABTS, antimicrobial activity, antibiofilm activity, antioxidant activity, cytotoxicity, DPPH, *Dracocephalum botryoides*, essential oil, GC–MS

## Abstract

*Dracocephalum botryoides* Stev. (Lamiaceae) is an endemic species of the Caucasus region with a history of traditional medicinal use, although the biological properties of its essential oil remain insufficiently characterized. In this study, the essential oil obtained from the aerial parts of *D. botryoides* collected in northern Azerbaijan was evaluated for its chemical composition, antioxidant, antimicrobial, antibiofilm, and cytotoxic activities. GC–MS analysis revealed a terpene-rich profile, with *p*-cymene (15.2%), T-cadinol (6.2%), caryophyllene oxide (6.0%), β-caryophyllene (5.8%), and sabinene (5.1%) as the major constituents. The essential oil showed notable antioxidant activity in DPPH and ABTS radical scavenging assays, with IC_50_ values of approximately 60 and 63 µg/mL, respectively. The essential oil exhibited antimicrobial activity, with minimum inhibitory concentration (MIC) values of 0.2% (*v*/*v*) against *Staphylococcus aureus* ATCC 29213, *Enterococcus faecalis* ATCC 29212, and *Candida albicans* ATCC 10231. Higher MIC values were recorded for *Escherichia coli* ATCC 25922 and *Candida glabrata* ATCC 2001 (1.0% *v*/*v*), while the highest MIC value was observed for *Trichophyton rubrum* ATCC 28188 (2.5% *v*/*v*). The essential oil also inhibited biofilm formation, and scanning electron microscopy supported these findings by demonstrating reduced biofilm coverage and disrupted biofilm architecture. In vitro assays using HaCaT human keratinocytes indicated low cytotoxicity of the essential oil at concentrations below 100 µg/mL. These results suggest that the terpene-rich essential oil of *D. botryoides* possesses noteworthy antioxidant, antimicrobial, and antibiofilm potential.

## 1. Introduction

The genus *Dracocephalum* L. (Lamiaceae) comprises approximately 60–70 species distributed throughout the temperate regions of the Northern Hemisphere, with particularly high species richness in Central Asia and the Caucasus. Species of this genus have long been used in traditional medicine and are recognized as valuable sources of essential oils (EOs) and structurally diverse secondary metabolites, including flavonoids, phenolic acids, terpenoids, and polysaccharides [1].

Four species of *Dracocephalum* have been recorded in the flora of Azerbaijan. Although three of these species have previously undergone partial pharmacognostic and phytochemical investigation, the available data remain limited. Recent studies have expanded the phytochemical knowledge of Azerbaijani *Dracocephalum* species, including the identification and structural elucidation of previously undescribed acacetin glycosides in *Dracocephalum austriacum* L. [2]. These findings further highlight the importance of continued research on endemic and regionally distributed Caucasian taxa.

Among these species, *Dracocephalum botryoides* Stev. is a Caucasus endemic distributed in alpine and subalpine meadows and has been reported from Azerbaijan, as well as parts of Georgia and southern Russia (Dagestan). Within Azerbaijan, the species is primarily found in mountainous regions, particularly in the Guba, Gusar, and Ordubad districts. The aerial parts of the plant have traditionally been consumed as herbal infusions for the treatment of gastrointestinal disorders, and its ethnomedicinal applications are supported by preliminary phytochemical studies reporting bioactive metabolites such as rosmarinic acid and caffeic acid with antioxidant and anti-inflammatory properties [1,3,4,5].

Despite its traditional relevance and growing scientific interest, the EO of *D. botryoides* remains largely unexplored. Previous investigations have mainly focused on polar extracts and non-volatile constituents of the plant, whereas the chemical composition and biological properties of its volatile fraction have not been comprehensively characterized. This is particularly relevant because EOs, owing to their terpene-rich and lipophilic nature, may exhibit distinct biological activities compared to non-volatile extracts, including enhanced membrane interaction and antimicrobial efficacy.

Microbial infections continue to represent a major global health burden, further exacerbated by the rapid emergence of antimicrobial resistance (AMR). In addition to classical resistance mechanisms, biofilm formation has emerged as a critical factor contributing to persistent and recurrent infections. Biofilms are structured microbial communities embedded in a self-produced extracellular polymeric substance (EPS) matrix, which significantly reduces the susceptibility of microorganisms to antimicrobial agents and host immune responses. As a result, biofilm-associated microorganisms can tolerate antimicrobial concentrations many times higher than those required to inhibit planktonic cells, rendering conventional therapies increasingly ineffective [6,7,8,9,10].

Conventional antibiotics, although highly effective against planktonic microorganisms, often fail to eradicate biofilm-associated infections due to limited penetration, altered metabolic states of microbial cells, and protective effects of the EPS matrix. Furthermore, the widespread and often inappropriate use of antibiotics has accelerated the development of resistant strains, highlighting the urgent need for alternative or complementary antimicrobial strategies. In this context, plant-derived EOs have gained increasing attention as promising candidates due to their complex chemical composition and multi-target mechanisms of action. EOs consist of diverse mixtures of terpenes and oxygenated compounds that can act on multiple cellular targets simultaneously, including disruption of cell membrane integrity, alteration of membrane permeability, inhibition of enzymatic systems, and interference with quorum sensing pathways involved in biofilm formation and maturation. Unlike conventional single-target antibiotics, these multi-faceted mechanisms may reduce the likelihood of resistance development and provide enhanced efficacy against biofilm-associated microorganisms [6,7,8,9].

In addition to their antimicrobial and antibiofilm potential, EOs are also recognized for their antioxidant properties, which may contribute to their overall biological activity and therapeutic relevance. However, their lipophilic and membrane-active nature also raises concerns regarding cytotoxicity at higher concentrations. Therefore, evaluation of cytotoxic effects of EOs in relevant human cell models, such as keratinocytes, is necessary to assess their safety and potential applicability, particularly in topical or dermatological formulations [11].

Accordingly, the aim of the present study was to provide a comprehensive evaluation of the EO obtained from the aerial parts of *D. botryoides* by characterizing its chemical composition and assessing its antioxidant, antimicrobial, antibiofilm, and cytotoxic properties, with particular emphasis on its potential as a multi-target agent against biofilm-associated microbial infections.

## 2. Results

### 2.1. Chemical Composition of the Essential Oil

The EO obtained from the aerial parts of *D. botryoides* had a yield of 0.15% (*v*/*w*, dry weight) and was analyzed by GC–MS, leading to the identification of 73 volatile constituents, representing 94.7% of the total oil composition (Table 1). The EO was predominantly composed of monoterpene hydrocarbons, oxygenated monoterpenes, and oxygenated sesquiterpenes.

The major constituents were *p*-cymene (15.2%), T-cadinol (6.2%), caryophyllene oxide (6.0%), β-caryophyllene (5.8%), and sabinene (5.1%). Other terpenoids detected at notable levels included α-thujene, germacrone, γ-terpinene, terpinen-4-ol, α-bisabolol, and germacrene-B.

**Figure 1:**
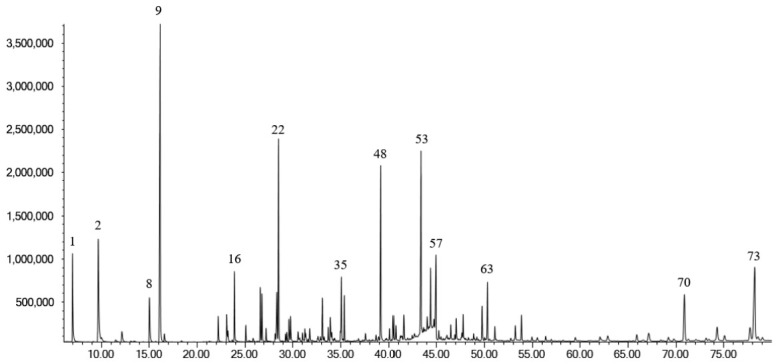
GC chromatogram of the EO of the aerial parts of *Dracocephalum botryoides*.

In addition to the identified compounds, nine unidentified constituents were detected in the EO. Among these, several occurred at relatively notable levels, indicating that the EO of *D. botryoides* may contain chemically interesting volatile metabolites that remain to be structurally characterized. The GC chromatogram of the EO is shown in Figure 1, while the mass spectra of unidentified constituents are provided in the Supplementary Materials.

To provide a clearer overview of the chemical profile of the EO, the identified constituents were further classified according to their chemical groups, and their distribution is summarized in Table 2.

### 2.2. Antimicrobial Activity of the Essential Oil

**Figure 2:**
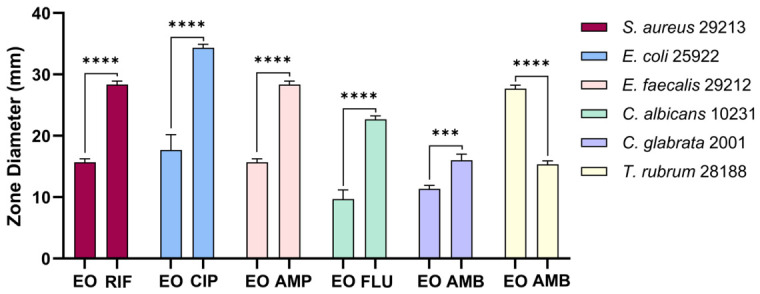
Antimicrobial activity of the EO of *D. botryoides*, evaluated by the disc diffusion assay and expressed as inhibition zone diameter (mm). Data are presented as mean ± SD (*n* = 3). Statistical comparisons between the EO-treated group and the corresponding positive control for each tested microorganism were performed using an independent two-tailed *t*-test (*** *p* < 0.001, **** *p* < 0.0001).

The antimicrobial activity of the *D. botryoides* EO was evaluated against selected bacterial, yeast, and dermatophyte strains using disc diffusion and broth microdilution methods. In the disc diffusion assay, the largest inhibition zone among the bacterial strains was observed against *E. coli* (17.67 ± 2.51 mm), followed by *S. aureus* and *E. faecalis* (15.66 ± 0.57 mm). Among the fungal strains, the highest inhibition zone was observed for *Candida glabrata* (11.33 ± 0.58 mm). Notably, the dermatophyte *Trichophyton rubrum* showed the highest inhibition zone overall (27.67 ± 0.58 mm) (Figure 2). The positive controls produced larger inhibition zones than the EO-treated discs for all tested microorganisms.

The minimum inhibitory concentration (MIC) values are presented in Table 3. The EO displayed the strongest inhibitory effect against *S. aureus* ATCC 29213, *E. faecalis* ATCC 29212, and *C. albicans* ATCC 10231, all with MIC values of 0.2% (*v*/*v*). Higher MIC values were recorded for *E. coli* ATCC 25922 and *C. glabrata* ATCC 2001 (1.0% *v*/*v*), while the highest MIC value was observed for *T. rubrum* ATCC 28188 (2.5% *v*/*v*). Based on these results, sub-inhibitory concentrations corresponding to 1/2 MIC were selected for subsequent antibiofilm assays against the selected test strains.

### 2.3. Antibiofilm Activity and SEM Imaging of Biofilm Structure

**Figure 3:**
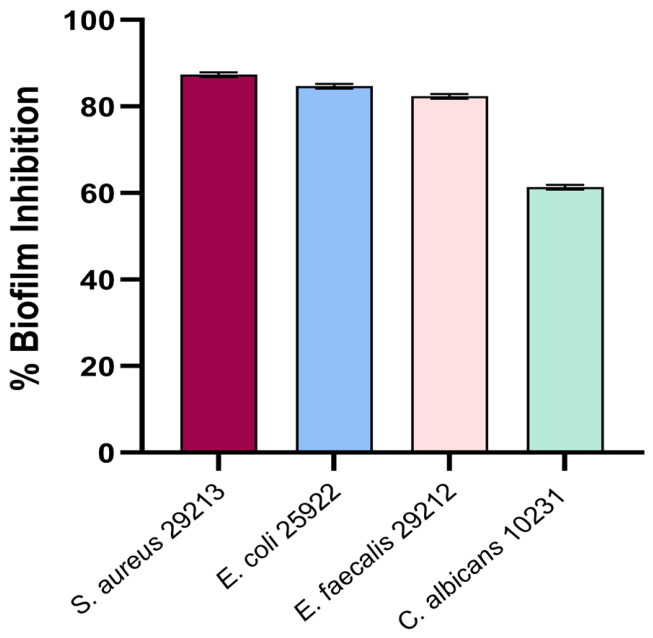
Antibiofilm activity of the EO expressed as percentage inhibition of biofilm formation. Data are presented as mean ± SD (*n* = 3). Statistical analysis was performed using one-way ANOVA followed by Tukey’s post hoc test. Statistical significance was set at *p* < 0.05.

Based on the MIC values (Table 3), sub-inhibitory concentrations corresponding to 1/2 MIC were selected to assess antibiofilm activity. The antibiofilm potential of the *D. botryoides* EO was evaluated using an in vitro biofilm formation assay against the tested microorganisms. The EO inhibited biofilm formation most strongly in *S. aureus* (87.33 ± 0.58%), followed by *E. coli* (84.67 ± 0.58%) and *E. faecalis* (82.33 ± 0.58%), whereas the lowest inhibition was observed for *C. albicans* (61.33 ± 0.58%). In the antibiofilm assay, chlorhexidine showed inhibition values of 85% against *S. aureus*, 87% against *E. coli*, 85% against *E. faecalis*, and 88% against *C. albicans*. One-way ANOVA indicated significant differences among the microorganisms (*p* < 0.0001), and Tukey’s post hoc test confirmed statistically significant pairwise differences (Figure 3).

**Figure 4:**
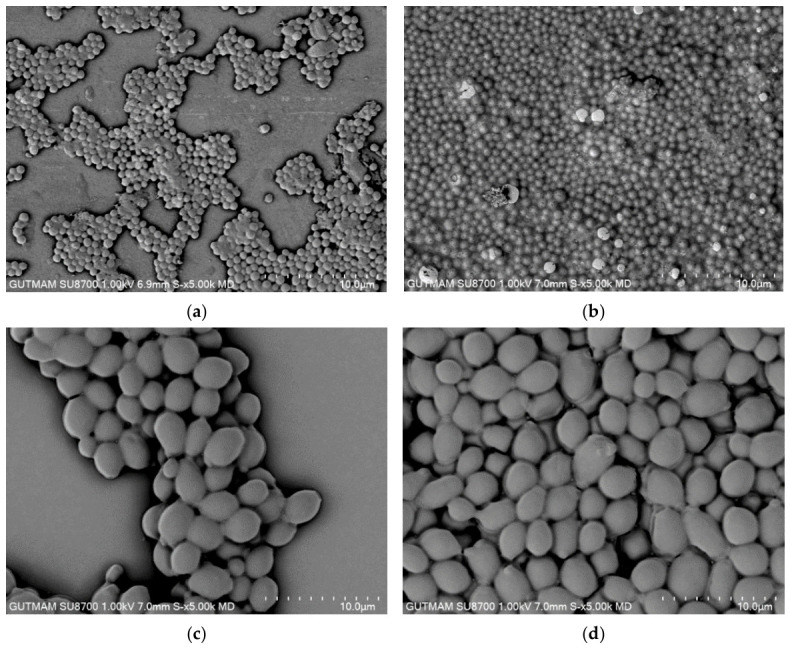
SEM micrographs of biofilms formed by *S. aureus* and *C. albicans* under control conditions and in the presence of the essential oil (EO) at sub-MIC: (**a**) *S. aureus* biofilm treated with EO; (**b**) untreated *S. aureus* biofilm; (**c**) *C. albicans* biofilm treated with EO; (**d**) untreated *C. albicans* biofilm.

To visualize the EO-induced changes in biofilm architecture, scanning electron microscopy (SEM) was performed on biofilms formed on glass coverslips in the presence of the corresponding sub-MICs. Compared with the untreated controls, EO-treated samples caused reduced biofilm coverage and altered biofilm structure (Figure 3 and Figure 4). Untreated control samples showed dense, well-developed biofilms extensively covering the surface, with microbial cells embedded in an extracellular polymeric substance (EPS)-like matrix. In contrast, treated samples exhibited irregular cell distribution, areas of partial detachment from the surface, and a less compact EPS matrix compared to those of the controls.

### 2.4. Cytotoxicity Assessment

**Figure 5:**
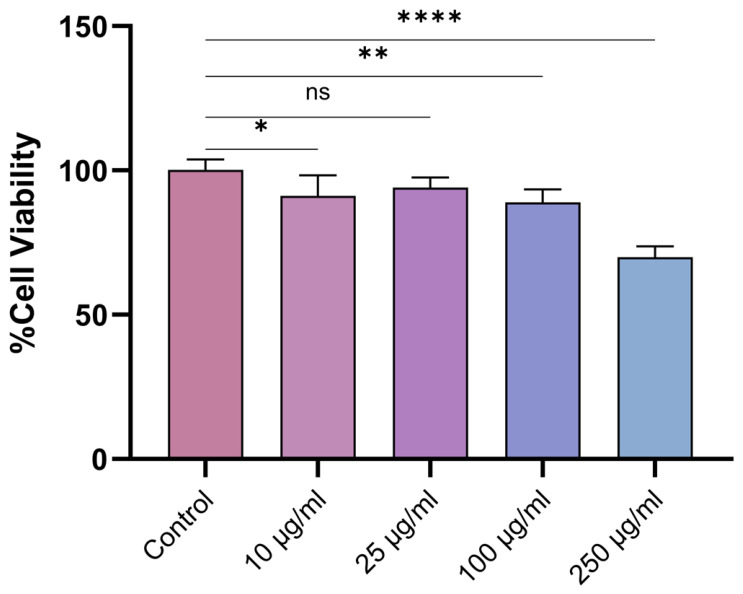
Cytotoxic effect of *D. botryoides* essential oil (EO) on HaCaT human keratinocyte cells determined by the MTT assay after 24 h of exposure. Results are presented as mean ± SD (*n* = 6). Statistical analysis was performed using one-way ANOVA followed by Dunnett’s post hoc test. Statistical significance is indicated as ns, not significant; * *p* < 0.05; ** *p* < 0.01; **** *p* < 0.0001.

The cytotoxicity of *D. botryoides* EO was evaluated using the MTT assay in HaCaT human keratinocyte cells. Cells were exposed to the EO at 10, 25, 100, and 250 µg/mL for 24 h. The EO was diluted in absolute ethanol, the final ethanol concentration in all wells was maintained at a non-cytotoxic level, and corresponding solvent controls were included. A concentration-dependent decrease in cell viability was observed (Figure 5).

At 10 µg/mL, cell viability remained high (91.2 ± 7.2%), and similarly low cytotoxicity was observed at 25 µg/mL (94.1 ± 3.4%). A moderate decrease in viability was detected at 100 µg/mL (88.9 ± 4.5%), whereas the most pronounced effect occurred at 250 µg/mL, where viability declined to 69.9 ± 3.8% (*p* < 0.05). Overall, these results indicate low cytotoxicity at concentrations up to 100 µg/mL under the applied experimental conditions.

### 2.5. Antioxidant Activity

The antioxidant activity of the EO was evaluated using DPPH and ABTS radical scavenging assays, and the results were expressed as percentage inhibition and IC_50_ values.

#### 2.5.1. DPPH Radical Scavenging Activity

The EO exhibited a concentration-dependent increase in DPPH radical scavenging activity. The percentage inhibition increased from approximately 8.2% at 25 µg/mL to 93.0% at 400 µg/mL. The IC_50_ value of the EO was approximately 60 µg/mL, indicating a moderate level of radical scavenging activity.

In contrast, ascorbic acid exhibited markedly stronger antioxidant activity, with inhibition values exceeding 90% even at the lowest tested concentration. Therefore, the IC_50_ value of ascorbic acid could not be accurately determined within the tested concentration range and was estimated to be below 25 µg/mL.

#### 2.5.2. ABTS Radical Scavenging Activity

Similar to the DPPH assay, the EO displayed a concentration-dependent increase in ABTS radical scavenging activity. The inhibition values ranged from approximately 11.2% at 25 µg/mL to 93.8% at 400 µg/mL. The IC_50_ value was approximately 63 µg/mL, which is consistent with the result obtained in the DPPH assay.

Ascorbic acid also demonstrated very strong ABTS radical scavenging activity, with inhibition values above 90% across all tested concentrations. Consequently, its IC_50_ value was estimated to be below 5 µg/mL.

#### 2.5.3. Comparative Evaluation

The results obtained from the DPPH and ABTS assays were in good agreement, with similar IC_50_ values observed for the EO in both test systems. This consistency supports the reliability of the antioxidant activity assessment. Overall, the EO exhibited moderate radical scavenging activity, whereas ascorbic acid was revealed to have markedly stronger antioxidant potential.

## 3. Discussion

The EO obtained from the aerial parts of *D. botryoides* showed a terpene-rich composition dominated by *p*-cymene, T-cadinol, caryophyllene oxide, β-caryophyllene, and sabinene. This general profile obtained in the current study is consistent with the known chemical characteristics of the genus *Dracocephalum*, whose EO are commonly rich in monoterpenes and sesquiterpenes [1,17,18,19]. In addition to the identified compounds, nine unidentified constituents were detected, indicating that the EO of *D. botryoides* may contain chemically interesting volatile metabolites. While the majority of the volatile profile was successfully characterized, a notable 6.0% of the EO consisted of a single unidentified constituent (unidentified-9). The presence of such a high concentration of an unknown compound in the endemic *D. botryoides* highlights its unique chemical diversity and suggests the presence of potentially novel metabolites that require further structural elucidation.

Comparison with the previous reports on *D. botryoides* EO [17] revealed notable differences in chemical composition of the EO. Compounds such as 1,3-cyclohexadiene derivatives, 3-octen-5-yne, and methyl isopropyl benzene, previously reported in Eos of the plant samples collected from other regions, were not detected in the present study [17]. In contrast, sabinene and *p*-cymene were among the predominant constituents of the present EO, whereas these compounds were reported to be absent or present only at trace levels in earlier work [17]. In addition, geraniol, reported as a major constituent in related species such as *D. moldavica*, was not detected in the present sample, further highlighting possibility of interspecific chemical variability within the genus [18]. These differences may be associated with geographical origin, altitude, local climatic conditions, phenological stage at the time of collection, and differences in extraction or analytical methodology [1,17,18].

The EO was demonstrated to possess notable antimicrobial activity. The strongest inhibitory effect was observed against the Gram-positive strains *S. aureus* and *E. faecalis*, with MIC values of 0.2% (*v*/*v*), whereas higher MIC values were obtained for *E. coli* and *C. albicans*. Similar antimicrobial effects have been reported for EOs from other *Dracocephalum* species [19]. A clear differential susceptibility was observed between Gram-positive and Gram-negative bacteria, with Gram-positive strains showing lower MIC values and stronger growth inhibition. This finding is consistent with well-established differences in bacterial cell envelope architecture. Gram-negative bacteria possess an outer membrane enriched in lipopolysaccharides, which constitutes an effective permeability barrier that limits the diffusion of hydrophobic compounds such as terpenoids. In contrast, Gram-positive bacteria lack this outer membrane, allowing easier access of hydrophobic EO constituents to the cytoplasmic membrane, where they may induce membrane destabilization, leakage of intracellular components, and impairment of essential cellular functions. Similar trends, which are considered a common, although not universal, phenomenon in EO research, have been widely reported for various EOs and are considered a common, although not universal, phenomenon in EO research [20,21,22,23].

**Figure 6:**
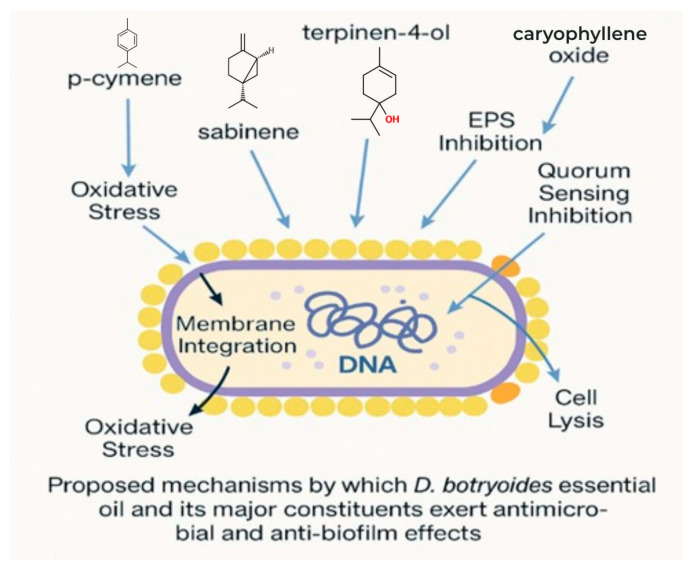
Proposed mechanisms underlying the antimicrobial and antibiofilm effects of *D. botryoides* EO. Dominant constituents (e.g., *p*-cymene, sabinene, terpinen-4-ol, and caryophyllene oxide) may contribute to these effects through combined actions, including interactions with microbial cell envelopes, modulation of oxidative stress, reduced extracellular polymeric substance (EPS) production, and interference with quorum-sensing-related processes, thereby impairing growth and biofilm development. In the current study, the crystal violet assay indicated high levels of biofilm inhibition by the EO, while SEM imaging revealed reduced surface coverage and altered biofilm architecture in EO-treated samples. Together, these findings support an EO-associated effect on biofilm establishment and/or structural integrity under the applied experimental conditions. Previous studies have also shown that chlorhexidine may alter biofilm structure and matrix composition, including effects on extracellular components that are important for biofilm integrity and persistence [25,26,27]. In addition, EOs have been reported to inhibit biofilm formation by disrupting early adhesion events, interfering with EPS production, and modulating quorum-sensing pathways involved in biofilm maturation [6,7,8,9,10].

The observed antimicrobial activity may be related to the terpene-rich composition of the EO. As complex natural mixtures, EOs often exert their biological effects through the combined contribution of multiple constituents rather than through the action of a single compound [20,21,22]. In the present EO, *p*-cymene was the predominant constituent, accompanied by oxygenated terpenes such as caryophyllene oxide and T-cadinol. The antimicrobial and antibiofilm activities observed for the *D. botryoides* EO are therefore likely attributable to the combined and potentially synergistic actions of its major and minor constituents rather than to a single dominant compound. Compounds such as *p*-cymene, β-caryophyllene, and caryophyllene oxide have been associated with antimicrobial activity, membrane interaction, and disruption of essential cellular functions in terpene-rich essential oils [20,21,22,23,24]. In particular, *p*-cymene, a hydrophobic monoterpene, is thought to integrate into lipid bilayers, contributing to membrane destabilization and potentially enhancing the activity of other bioactive constituents rather than exerting strong antimicrobial effects on its own [20,22,24]. Although membrane integrity was not directly measured in the present study, the observed activity is consistent with these general mechanisms proposed for many EO constituents [20,21,22]. These proposed mechanisms are summarized schematically in Figure 6.

Beyond planktonic growth inhibition, the EO demonstrated strong antibiofilm activity when evaluated using the crystal violet (CV) assay after 24 h of incubation. The CV assay quantifies total biofilm biomass, including both microbial cells and extracellular polymeric substances (EPS), and is widely accepted as a standard method for assessing biofilm development. In contrast, anti-adhesion assays typically involve much shorter incubation periods and focus exclusively on initial surface attachment events. Therefore, the effects observed in the present study should be interpreted as suppression of biofilm formation during the early stages of biofilm establishment, including microcolony formation and matrix accumulation [28,29].

The decision to evaluate antibiofilm activity at sub-MICs was deliberate and mechanistically motivated. Sub-inhibitory exposure minimizes confounding effects related to growth inhibition and allows assessment of biofilm-associated virulence modulation. Increasing evidence indicates that EOs can interfere with quorum sensing, motility, EPS production, and other regulatory pathways critical for biofilm development, even at concentrations that do not fully inhibit planktonic growth. Such antivirulence-oriented effects are particularly attractive from a therapeutic perspective, as they may reduce selective pressure for resistance development while attenuating pathogenic behaviour [9,23].

The antioxidant activity observed in the DPPH and ABTS radical scavenging assays, with IC_50_ values of approximately 60 and 63 µg/mL, respectively, indicates that the EO possesses moderate radical scavenging capacity. In addition to its antimicrobial and antibiofilm effects, the EO of *D. botryoides* therefore also showed antioxidant activity in two complementary test systems. The similar IC_50_ values obtained in both assays suggest a consistent radical scavenging effect. Although the antioxidant activity was lower than that of ascorbic acid, the observed effect is relevant for a terpene-rich EO, which may result from the combined and possibly synergistic action of multiple volatile constituents. EOs are increasingly recognized not only for their antimicrobial properties but also for their antioxidant potential, which may enhance their biological and pharmacological significance [20,21,22]. In this context, the antioxidant findings further broaden the biological profile of the *D. botryoides* EO and support its phytopharmacological relevance.

Antibiofilm activity was assessed at a single time point (24 h) and at a single sub-MIC, focusing on early biofilm formation rather than mature biofilm disruption. Future studies incorporating concentration–response analyses, extended incubation times, and molecular investigations of quorum-sensing- and EPS-related pathways would provide deeper mechanistic insight. In addition, while several unidentified volatile constituents were detected in the EO, their structures and biological contributions remain to be elucidated. Despite these limitations, the present findings provide a robust experimental framework and a mechanistically informed basis for further exploration of *D. botryoides* EO as a biofilm-modulating and antimicrobial agent. The quantitative reduction in biofilm biomass measured by the crystal violet assay (ranging from 61% to 87%) was visually confirmed by SEM analysis, which revealed a significant depletion of extracellular matrix and a reduction in microcolony formation.

Cytotoxicity testing of the EO in HaCaT human keratinocyte cells indicated low cytotoxicity at concentrations up to 100 µg/mL, whereas a more pronounced reduction in viability was observed at 250 µg/mL. These findings suggest a relatively favourable in vitro biocompatibility profile within the lower concentration range tested. This interpretation is also consistent with previous studies evaluating the in vitro biological activities of essential oils in human skin cells [11]. Importantly, antibiofilm effects were observed at sub-MIC levels, supporting the existence of a practical safety window for topical or surface-associated applications. This is particularly relevant in antibiofilm strategies, where complete microbial eradication may not be required if virulence and biofilm formation are effectively suppressed. The lipophilic nature of EOs facilitates their interaction with microbial membranes, and their antimicrobial activity is generally concentration-dependent [20,21,22]. Their relative selectivity toward microbial cells has been partly attributed to differences in membrane composition between microbial and mammalian cells [20].

Nevertheless, some limitations should be acknowledged. Antibiofilm activity was assessed at a single time point (24 h) and at a single sub-MIC level, focusing on early biofilm formation rather than mature biofilm disruption. In addition, several unidentified volatile constituents were detected in the EO, and their structures and biological contributions remain to be elucidated. Future studies incorporating concentration-response analyses, extended incubation periods, mature biofilm models, and mechanistic assays related to quorum sensing, membrane integrity, and EPS production would provide deeper insight into the biological effects of this EO. Broader cytotoxicity testing in additional cell models and expanded evaluation against clinically relevant isolates would also help to better define the scope and limitations of its bioactivity.

## 4. Materials and Methods

### 4.1. Plant Collection and Essential Oil Extraction

The aerial parts of *D. botryoides* were collected during the flowering period from alpine meadows near Khinalig, Guba region, Azerbaijan (June 2020; 41°19′24.9″ N, 48°12′07.0″ E; 2850 m a.s.l.). The plant material was taxonomically identified by Prof. Dr. Javanshir Isayev (Department of Pharmacognosy, Azerbaijan Medical University) based on comparison with herbarium specimens of the Institute of Botany, Ministry of Science and Education of the Republic of Azerbaijan. A voucher specimen (No: Az/LDR-0720/87-114) was deposited in the Department of Pharmacognosy, Azerbaijan Medical University. The plant material was dried in a ventilated oven at 40 °C for 8–10 days and then stored at 4 °C until further use. The EO was obtained by hydrodistillation for 3 h using a Clevenger-type apparatus, following European Pharmacopoeia guidelines to avoid artefact formation. The EO yield was expressed as a percentage (*v*/*w*) relative to the dry weight of the plant material. The resulting EO was stored in amber glass vials at +4 °C. For chromatographic analysis, a stock solution was prepared using *n*-hexane as the solvent.

### 4.2. GC–MS Analysis

The chemical composition of the EO was analyzed by gas chromatography–mass spectrometry (GC–MS) according to previously described procedures, with minor modifications [17]. Analyses were performed on an Agilent 5975 GC–MSD system equipped with an HP-Innowax FSC capillary column (60 m × 0.25 mm i.d., 0.25 μm film thickness). Helium was used as the carrier gas at a flow rate of 0.8 mL/min. The oven temperature was programmed from 60 °C (10 min) to 220 °C at 4 °C/min and held for 10 min, then increased to 240 °C at 1 °C/min. The injector temperature was set at 250 °C, and samples were injected in split mode (40:1). Mass spectra were recorded in electron impact (EI) mode at 70 eV over an *m*/*z* range of 35–450.

### 4.3. Compound Identification

The chemical profile of the EO was identified by comparing the mass spectra of the constituents with those reported in the Wiley, NIST, MassFinder 3.0, Adams, and in-house Başer EO libraries, and by evaluating their relative retention indices (RRIs), calculated using a homologous series of *n*-alkanes (C8–C40). Relative percentages of the identified compounds were calculated based on GC–MS total ion chromatogram (TIC) peak area normalization.

### 4.4. Microorganisms and Antimicrobial Activity

The EO was tested against *S. aureus* ATCC 29213, *E. coli* ATCC 25922, *E. faecalis* ATCC 29212, *C. albicans* ATCC 10231, *C. glabrata* ATCC 2001, and *T. rubrum* ATCC 28188. All strains were obtained from the American Type Culture Collection (ATCC, Manassas, VA, USA).

The antimicrobial activity of the EO was evaluated using the disc diffusion method described by Kirby–Bauer [30], while the broth microdilution assay was performed in accordance with Clinical and Laboratory Standards Institute (CLSI) guidelines [31].

Before each experiment, bacterial strains were subcultured on Mueller–Hinton agar (MHA) and incubated at 37 °C for 24 h. *Candida* species were cultured on Sabouraud dextrose agar (SDA) and incubated at 35–37 °C for 24–48 h. *T. rubrum* ATCC 28188 was maintained on SDA and incubated at 28–30 °C for 5–7 days until sufficient sporulation and mycelial growth were achieved. Fresh cultures were used for all assays. Bacterial and yeast inocula were prepared by suspending colonies in sterile saline and adjusting turbidity to 0.5 McFarland standard using a densitometer (Grant Bio DEN-1, Ankara, Türkiye). For *T. rubrum*, the inoculum was prepared by harvesting conidia and hyphal fragments from actively growing cultures in sterile saline supplemented with 0.05% (*v*/*v*) Tween 80. The suspension was homogenized, and large hyphal aggregates were allowed to settle to obtain a uniform inoculum.

For the disc diffusion assay, 100 µL of each standardized inoculum was spread onto the appropriate agar medium. MHA was used for bacterial strains, while SDA was used for *Candida* spp. and *T. rubrum.* Sterile 6 mm paper discs (Bioanalyse, Ankara, Türkiye) were placed on the agar surface and loaded with 20 µL of the EO. Plates inoculated with bacteria were incubated at 37 °C for 24 h, and *Candida* spp. at 35–37 °C for 24–48 h, and *T. rubrum* at 28–30 °C until visible growth was obtained. The diameters of inhibition zones were measured in millimetres, including the disc diameter. All experiments were performed in triplicate, and results were expressed as mean ± standard deviation (SD).

The following antimicrobial discs were used as positive controls: ciprofloxacin (CIP, 5 µg/disc) for *E. coli* ATCC 25922, rifampicin (RIF, 5 µg/disc) for *S. aureus* ATCC 29213, and ampicillin (AMP, 10 µg/disc) for *E. faecalis* ATCC 29212. For the antifungal assays, fluconazole (FLU, 25 µg/disc) was used as the positive control for *C. albicans* ATCC 10231, whereas amphotericin B (AMB, 100 µg/disc) was used for *C. glabrata* ATCC 2001 and *T. rubrum* ATCC 28188.

The MIC values were determined using the broth microdilution method in sterile 96-well microplates following CLSI recommendations. EO stock solutions were prepared in dimethyl sulfoxide (DMSO) and serially diluted in the appropriate broth medium using a two-fold dilution scheme. The final DMSO concentration did not exceed 1% (*v*/*v*) and was verified to not affect microbial growth. For bacterial strains, the assay was conducted in suitable bacterial broth medium, while Sabouraud dextrose broth (SDB) was used for *Candida* spp. and *T. rubrum*, in accordance with CLSI antifungal susceptibility testing guidelines. Each well was inoculated with standardized microbial suspension, and the plates included growth control (medium + inoculum) and sterility control (medium only) wells. Microplates were incubated at 37 °C for 24 h for bacteria, 35–37 °C for 24–48 h for *Candida* spp., and 28–30 °C for *T. rubrum* until visible growth was observed. The MIC was defined as the lowest EO concentration at which no visible microbial growth was detected. All assays were performed in triplicate.

### 4.5. Antibiofilm Assay and SEM Imaging

The antibiofilm activity of the EO was evaluated using the crystal violet (CV) staining method [28]. Test microorganisms were cultured in the appropriate growth media, as described in the antimicrobial activity assay, and incubated at 37 °C for 24 h. Following incubation, microbial suspensions were adjusted to a 0.5 McFarland standard. Aliquots of the standardized inocula were transferred into 96-well microtiter plates containing sub-MICs of the EO and the corresponding growth medium, and the plates were incubated at 37 °C for an additional 24 h to allow biofilm formation. The wells containing microbial cultures without the EO served as untreated controls. Chlorhexidine gluconate (CHX) (20%; Chem Pure, 1 L; ZAG Kimya, Istanbul, Türkiye) was used as a positive control in the antibiofilm assay due to its well-established broad-spectrum antimicrobial activity and its reported efficacy against both bacterial and fungal biofilms [25,26,29]. After incubation, the culture medium was carefully removed, and the wells were gently washed three times with phosphate-buffered saline (PBS) to remove non-adherent cells. The plates were then air-dried at 65 °C and stained with crystal violet solution for 30 min. Excess dye was removed, and the wells were rinsed with PBS. The bound CV was subsequently solubilized using acetic acid, and the absorbance was measured at 595 nm using a microplate reader (BMG Labtech, CLARIOstar, Ortenberg, Germany). All experiments were performed in triplicate. Biofilm inhibition was calculated using the following formula:

Inhibition (%) = [(OD_595_ control − OD_595_ sample)/OD_595_ control] × 100

where OD_595_ control represents the absorbance of the untreated control biofilm and OD_595_ sample represents the absorbance of the biofilm treated with sub-MICs of the EO. Here, OD stands for optical density (i.e., the absorbance value measured by the microplate reader at the indicated wavelength).

The antibiofilm effect of the EO was further visualized using scanning electron microscopy (SEM). Test microorganisms were cultured in the appropriate growth medium at 37 °C for 24 h. Sterile glass coverslips were placed in 6-well tissue culture plates, and microbial suspensions adjusted to a 0.5 McFarland standard were added to each well along with sub-MICs of the EO. After incubation, the culture medium and non-adherent cells were carefully removed. The biofilm-coated coverslips were gently washed with 0.01 M PBS and fixed with 2.5% (*v*/*v*) glutaraldehyde. Biofilms were dehydrated through a graded ethanol series (30%, 50%, 60%, 70%, 80%, 90%, 95%, and 100%), air-dried, sputter-coated with gold, and examined using a high-vacuum scanning electron microscope (Hitachi SU5000, Ankara, Türkiye).

### 4.6. Cytotoxicity Assay

The cytotoxicity of the EO was evaluated using the MTT assay in the human keratinocyte cell line HaCaT (ATCC^®^) to assess biocompatibility [32]. Cells were seeded into 96-well plates at a density of 2 × 10^4^ cells per well and allowed to adhere overnight. The EO was first dissolved in absolute ethanol and then diluted to the required final concentrations (10, 25, 100, and 250 µg/mL) in the culture medium. The final ethanol concentration in all wells was maintained at a non-cytotoxic level, and corresponding solvent controls were included in the assay. Cells were incubated with the EO for 24 h. Untreated cells cultured in complete medium served as the negative control (100% viability). After treatment, 100 µL of MTT solution (1 mg/mL) was added to each well, and the plates were incubated for 4 h at 37 °C to allow the formation of formazan crystals in live cells. The resulting crystals were solubilized using acidified isopropanol solution (0.04 M HCl in isopropanol), and absorbance was measured at 570 nm using a microplate reader (CLARIOstar, BMG Labtech, Ortenberg, Germany). Cell viability (%) was calculated as follows:

Cell viability (%) = (OD_570_ sample/OD_570_ control) × 100

where OD_570_ represents the optical density (absorbance) measured at 570 nm, OD_570_ (sample) represents the absorbance of EO-treated cells, and OD_570_ (control) represents the absorbance of untreated control cells (100% viability). Results are expressed as mean ± SD (*n* = 6).

### 4.7. Antioxidant Activity Assays

The antioxidant activity of the EO was evaluated using two complementary radical scavenging assays, namely DPPH and ABTS, according to previously reported methods with slight modifications [33,34]. Ascorbic acid was used as the reference antioxidant in both assays.

#### 4.7.1. DPPH Radical Scavenging Assay

The free radical scavenging activity of the EO was determined using the DPPH assay according to the method of Brand-Williams et al., with minor modifications [33]. Stock solutions of the EO and ascorbic acid were prepared in methanol. The EO was tested at concentrations of 25, 50, 100, 200, and 400 µg/mL.

In a 96-well microplate, 200 µL of each sample solution was mixed with 50 µL of freshly prepared 1 mM DPPH solution in methanol. Methanol containing DPPH without sample was used as the control. The reaction mixtures were incubated in the dark at room temperature for 30 min, and the absorbance was measured at 517 nm using a microplate reader. All measurements were performed in quadruplicate.

The percentage of DPPH radical scavenging activity was calculated using the following equation:

DPPH inhibition (%) = [(A_control_ − A_sample_)/A_control_] × 100

where A_control_ is the absorbance of the control reaction and A_sample_ is the absorbance of the tested sample.

#### 4.7.2. ABTS Radical Scavenging Assay

The ABTS radical scavenging activity was determined according to the method described by Re et al., with slight modifications [34]. The ABTS stock solution was prepared by dissolving 38.6 mg of ABTS in 10 mL of distilled water. Potassium persulfate solution (6.6 mg/10 mL) was used as the oxidizing agent. The ABTS radical cation (ABTS^•+^) was generated by mixing equal volumes of ABTS and potassium persulfate solutions and allowing the mixture to stand in the dark at room temperature for 16 h.

Before use, the ABTS^•+^ solution was diluted with methanol to obtain a working solution with an absorbance of 0.70 ± 0.02 at 734 nm. The EO was tested at concentrations of 25, 50, 100, 200, and 400 µg/mL, whereas ascorbic acid was tested at concentrations of 5, 10, 25, 50, and 100 µg/mL.

In a 96-well microplate, the sample solutions were mixed with the ABTS working solution and incubated at room temperature in the dark. The absorbance was then measured at 734 nm using a microplate reader. All experiments were carried out in quadruplicate.

The percentage of ABTS radical scavenging activity was calculated using the following equation:

ABTS inhibition (%) = [(A_control_ − A_sample_)/A_control_] × 100

where A_control_ is the absorbance of the control reaction and A_sample_ is the absorbance of the tested sample.

#### 4.7.3. IC_50_ Determination

The IC_50_ values, defined as the concentration required to inhibit 50% of the radicals, were calculated from the concentration–response curves using nonlinear regression analysis.

### 4.8. Statistical Analysis

Antimicrobial and antibiofilm assays were performed in triplicate (*n* = 3), while cytotoxicity experiments were conducted in sextuplicate (*n* = 6). Data are presented as mean ± standard deviation (SD). Statistical analyses were performed using GraphPad Prism software (version 10.3.1, GraphPad Software, San Diego, CA, USA).

One-way analysis of variance (ANOVA) was used to evaluate differences among multiple groups, including inhibition zone diameters, biofilm inhibition percentages, and cell viability data. Tukey’s honestly significant difference (HSD) post hoc test was applied for pairwise comparisons between all groups to assess concentration-dependent effects, while Dunnett’s post hoc test was used to compare treatment groups with the corresponding control.

A *p*-value of less than 0.05 was considered statistically significant. Statistical significance was defined as follows: ns, *p* > 0.05; * *p* < 0.05; ** *p* < 0.01; *** *p* < 0.001; **** *p* < 0.0001. Statistical significance indicators are presented in Figure 2, Figure 3 and Figure 5.

## 5. Conclusions

Our findings revealed that the EO of *D. botryoides* collected in northern Azerbaijan showed a terpene-rich GC–MS profile dominated by *p*-cymene together with oxygenated terpenes. The chemical analysis identified a unique profile dominated by *p*-cymene and oxygenated sesquiterpenes, which likely drive the observed bioactivities. The EO exhibited moderate antioxidant activity in DPPH and ABTS radical scavenging assays, having IC_50_ values of approximately 60 and 63 µg/mL, respectively. In addition, the EO showed antimicrobial activity, with the lowest MIC values against *S. aureus*, *E. faecalis*, and *C. albicans* (0.2% *v*/*v*), while higher MIC values were observed for *E. coli* and *C. glabrata* (1.0% *v*/*v*) and the highest value for *T. rubrum* (2.5% *v*/*v*). At sub-inhibitory levels (1/2 MIC), the EO inhibited biofilm formation, and SEM imaging supported these findings by revealing reduced biofilm coverage and altered biofilm architecture in treated samples. In HaCaT keratinocyte cells, the EO displayed low cytotoxicity up to 100 µg/mL under the applied conditions, whereas higher concentrations produced a more pronounced decrease in viability. The absence of significant cytotoxicity toward human keratinocytes (HaCaT cells) at bioactive levels highlights a favourable safety of the EO. Overall, these findings provide an integrated characterization of the chemical composition and biological profile of *D. botryoides* EO. Future investigations should focus on the specific molecular mechanisms of its antibiofilm action and the potential synergistic contributions of its unidentified volatile constituents in in vivo settings.

## Figures and Tables

**Table 1:** Volatile constituents identified by GC–MS in the EO of *D. botryoides* and their corresponding relative retention indices (RRIs).

No	RRI_Exp_	RRI_Lit_	Compound Name *	Content (%)	Reference
**1**	1035	1012–1039	**α-Thujene**	**3.1**	[12]
**2**	1132	1098–1140	**Sabinene**	**5.1**	[12]
3	1135	1109–1137	Thuja-2,4(10)-diene	0.3	[12]
4	1174	1140–1175	Myrcene	0.2	[12]
5	1188	1154–1195	α-Terpinene	0.6	[12]
6	1203	1178–1219	Limonene	0.1	[12]
7	1218	1188–1233	β-Phellandrene	0.1	[12]
**8**	1255	1222–1266	**γ-Terpinene**	**2.0**	[12]
**9**	1280	1246–1291	***p*-Cymene**	**15.2**	[12]
10	1290	1261–1300	Terpinolene	0.4	[12]
11	1330	1339	Pinol	0.1	[13]
12	1393	1378–1408	3-Octanol	0.1	[12]
13	1435	-	*Unidentified-1*	0.9	-
14	1441	1434–1441	β-Thujone	0.9	[14]
15	1452	1411–1465	1-Octen-3-ol	0.5	[12]
**16**	1474	1469–1476	***trans*-Sabinene hydrate**	**2.2**	[14]
17	1530	-	*Unidentified-2*	0.5	-
18	1553	1507–1564	Linalool	1.4	[12]
19	1556	1526–1565	*cis*-Sabinene hydrate	1.4	[12]
20	1575	1557–1625	*trans*-*p*-Menth-2-en-1-ol	0.4	[12]
21	1611	1564–1630	Terpinen-4-ol	1.8	[12]
**22**	1612	1569–1632	**β-Caryophyllene**	**5.8**	[12]
23	1642	1641–1642	Thuj-3-en-10-al	0.2	[12]
24	1650	1612–1654	γ-Elemene	0.7	[12]
25	1651	1606–1683	Sabinaketone	0.3	[12]
26	1657	1610–1667	Umbellulone	0.7	[12]
27	1685	1683–1720	*trans*-Sabinol	0.3	[12]
28	1687	1680–1705	α-Humulene	0.2	[12]
29	1693	1674–1708	β-Acoradiene	0.3	[12]
30	1706	1659–1724	α-Terpineol	0.3	[12]
31	1726	1676–1726	Germacrene D	0.5	[12]
32	1758	1668–1771	*cis*-Piperitol	0.1	[12]
33	1765	1728–1772	Geranyl acetate	0.2	[12]
34	1776	1735–1782	γ-Cadinene	1.5	[12]
35	1797	1739–1797	*p*-Methyl acetophenone	0.1	[12]
36	1802	1747–1805	Cuminaldehyde	0.9	[12]
37	1811	1790–1821	*p*-Mentha-1(7),8-dien-2-ol	0.2	[14]
38	1814	1803–1815	*p*-Mentha-1,5-dien-7-ol	0.5	[14]
39	1820	-	*epi*-Shyobunol acetate ^#^	0.4	[12]
40	1823	1774–1821	*trans-p*-Mentha-1(7),5-dien-2-ol	0.2	[12]
**41**	1854	1778–1854	**Germacrene-B**	**2.2**	[12]
42	1865	1813–1865	*p*-Cymen-8-ol	0.3	[12]
43	1868	1820–1873	(*E*)-Geranylacetone	0.2	[12]
44	1870	-	Pregeijerene ^§^	1.2	[12]
45	1940	1940	4-Isopropyl salicylaldehyde	0.4	[15]
46	1988	1984	8,9-Dehydrothymol	0.1	[16]
47	2001	1976–2003	*iso*-Caryophyllene oxide	0.1	[14]
**48**	2008	1936–2023	**Caryophyllene oxide**	**6.0**	[12]
49	2073	2065–2073	*p*-Mentha-1,4-dien-7-ol	1.1	[12]
50	2080	2019–2090	Cubenol	0.6	[12]
51	2107	2091–2107	β-Elemenone	1.0	[12]
52	2113	2078–2115	Cumin alcohol	0.1	[14]
**53**	2187	2148–2200	**T-Cadinol**	**6.2**	[12]
54	2198	2100–2205	Thymol	0.4	[12]
55	2232	2178–2234	α-Bisabolol	1.5	[12]
56	2250	2180–2255	α-Cadinol	0.2	[12]
**57**	2264	2209–2254	**Germacrone**	**3.2**	[14]
58	2270	2262–2269	Guaia-6,10(14)-dien-4 beta-ol	0.3	[12]
59	2320	2324	14-Nor-cadin-5-en-4-one *isomer A*	0.4	[12]
60	2340	2349	Cadina-4,10(15)-dien-3-one	0.7	[14]
61	2392	2371–2405	Caryophyllenol-II	0.7	[12]
62	2503	-	*Unidentified-3*	1.2	-
**63**	2524	2476–2523	**Abietatriene**	**2.0**	[12]
64	2620	2510–2633	Phytol	0.2	[12]
65	2638	-	*Unidentified-4*	0.5	-
66	2685	-	*Unidentified-5*	0.9	-
67	2700	2700	Heptacosane	0.4	[14]
68	2896	2862–2945	Hexadecanoic acid	0.4	[12]
69	2900	2900	Nonacosane	0.3	[14]
**70**	3103	-	***Unidentified-6***	**3.2**	-
71	3167	-	*Unidentified-7*	1.0	-
72	3230	-	*Unidentified-8*	1.0	-
**73**	3239	-	***Unidentified-9***	**6.0**	-
			**Total**	**94.7**	

*: ≥0.1%. ^#^: Tentative identification from Wiley-NIST similarity search. ^§^: Correct isomer not identified. Bold indicates major compounds. Italics indicate unidentified compounds.

**Table 2:** Distribution of the identified volatile constituents of the essential oil of *D. botryoides* according to chemical classes.

**Monoterpene hydrocarbons**	27.1
**Oxygenated monoterpenes**	19.6
**Sesquiterpene hydrocarbons**	7.2
**Oxygenated sesquiterpenes**	22.3
**Diterpenes**	3.2
**Oxygenated diterpenes**	8.2
**Aliphatic alcohols**	0.6
**Aliphatic ketones**	0.2
**Aromatic ketones**	0.4
**Aromatic hydrocarbons**	4.8
**Alkanes**	0.7
**Fatty acids**	0.4

**Table 3:** Minimum inhibitory concentrations (MICs) of the EO of *D. botryoides* against the tested microorganisms and the corresponding sub-MIC levels used for antibiofilm assays. Values are expressed as % (*v*/*v*).

Microorganisms	MIC (% *v*/*v*)	Sub-MIC (1/2 MIC) (% *v*/*v*)
*S. aureus* ATCC 29213	0.2	0.1
*E. faecalis* ATCC 29212	0.2	0.1
*E. coli* ATCC 25922	1.0	0.5
*C. albicans* ATCC 10231	0.2	0.1
*C. glabrata* ATCC 2001	1.0	0.5
*T. rubrum* ATCC 28188	2.5	1.25

## Data Availability

The data presented in this study are available from the corresponding author upon reasonable request.

## Supplementary Materials

The following supporting information can be downloaded at: https://www.mdpi.com/article/10.3390/plants15091416/s1, Figure S1: GC chromatogram of the essential oil of *Dracocephalum botryoides*; Figures S2–S10: Mass spectra of unidentified constituents detected in the essential oil of *D. botryoides*.
