# Supplementary material for: Chemical Composition, Antimicrobial, Antibiofilm Activities, and Cytotoxicity of the Essential Oil of Dracocephalum botryoides Stev
Source: Plants (Basel). 2026 May 6;15(9):1416. doi: 10.3390/plants15091416 (PMC13164927; doi:10.3390/plants15091416)

Supplementary Materials

Figure S1: GC chromatogram of the essential oil of *Dracocephalum botryoides*.

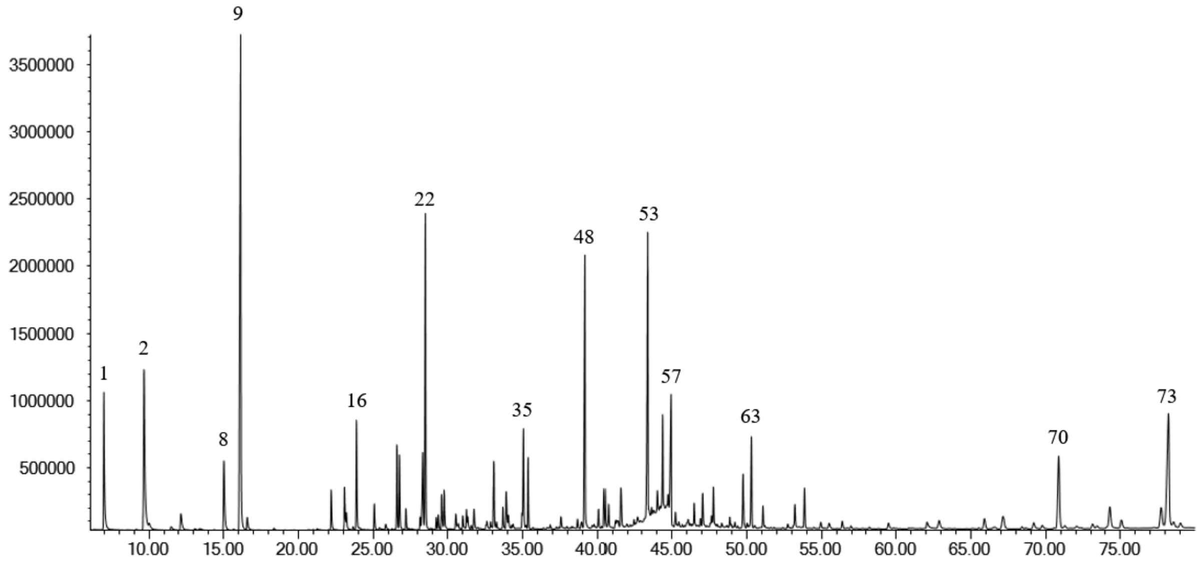

Unidentified Peaks

Figure S2: Mass spectrum of Unidentified-1 detected in the essential oil of *D. botryoides* (RRI_{Exp}: 1435; relative content: 0.9%).

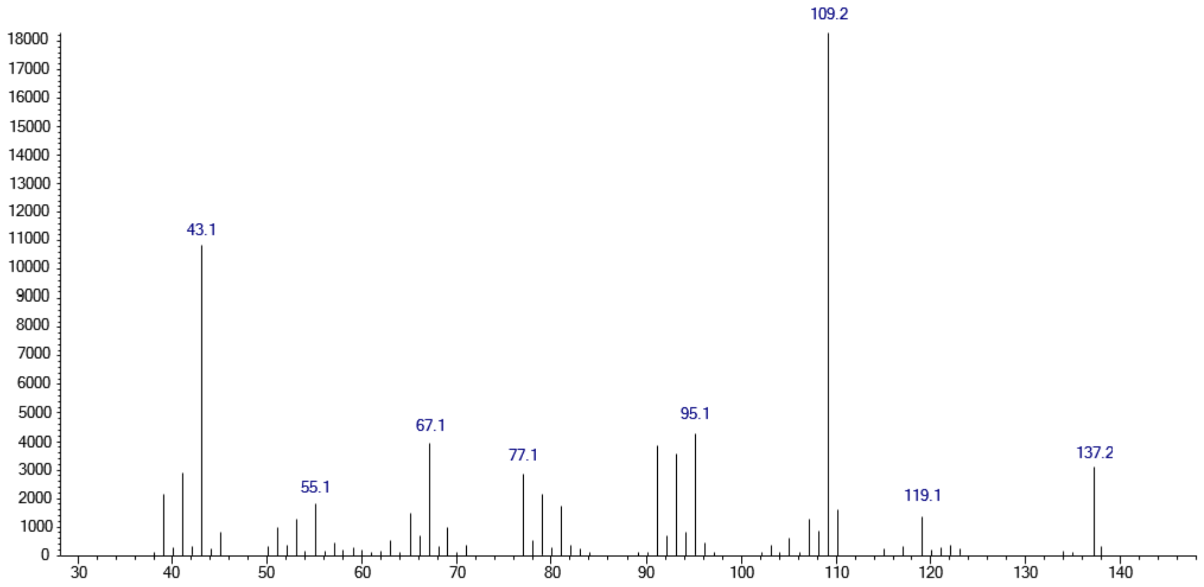

Supplementary Materials

Figure S3: Mass spectrum of Unidentified-2 detected in the essential oil of *D. botryoides* (RRI$_{Exp}$: 1530; relative content: 0.5%).

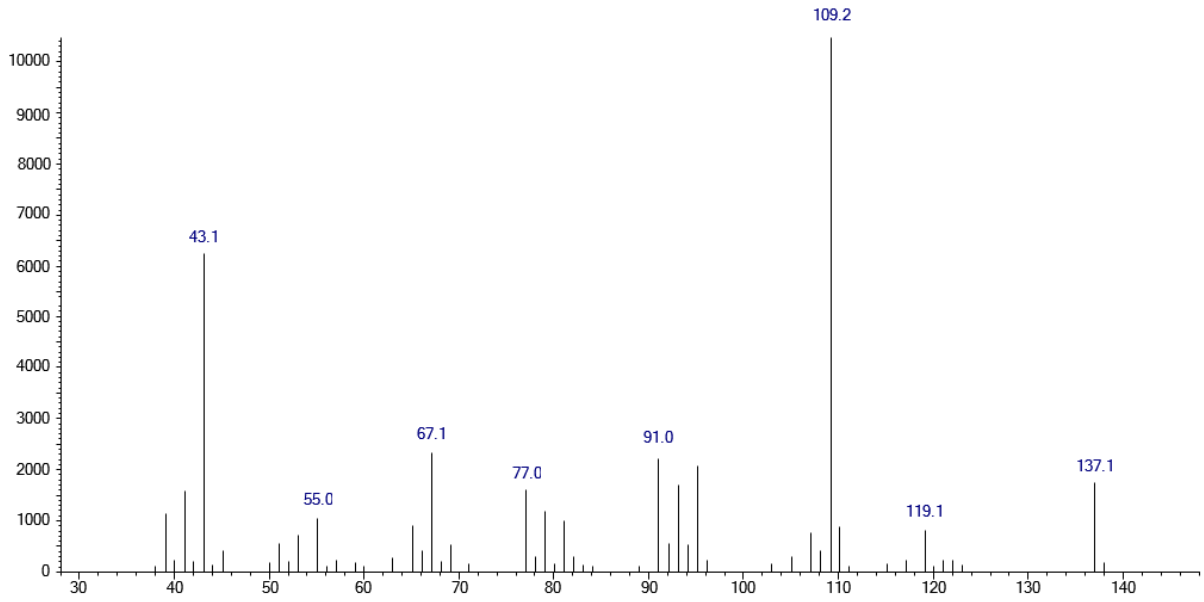

Figure S4: Mass spectrum of Unidentified-3 detected in the essential oil of *D. botryoides* (RRI$_{Exp}$: 2503; relative content: 1.2%).

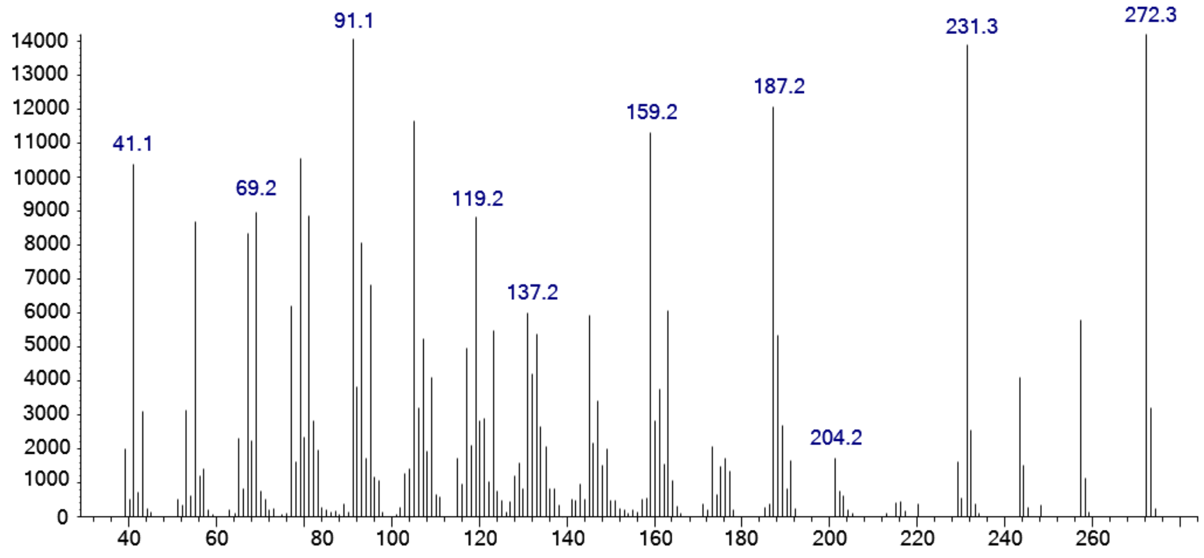

Supplementary Materials

Figure S5: Mass spectrum of Unidentified-4 detected in the essential oil of *D. botryoides* (RRI$_{Exp}$: 2638; relative content: 0.5%).

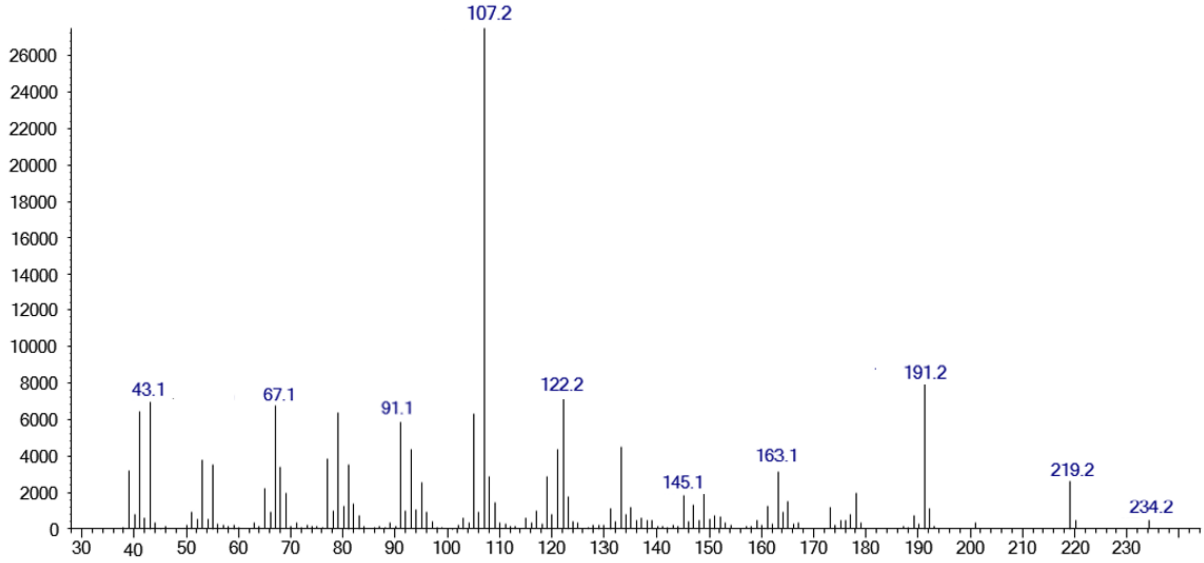

Figure S6: Mass spectrum of Unidentified-5 detected in the essential oil of *D. botryoides* (RRI$_{Exp}$: 2685; relative content: 0.9%).

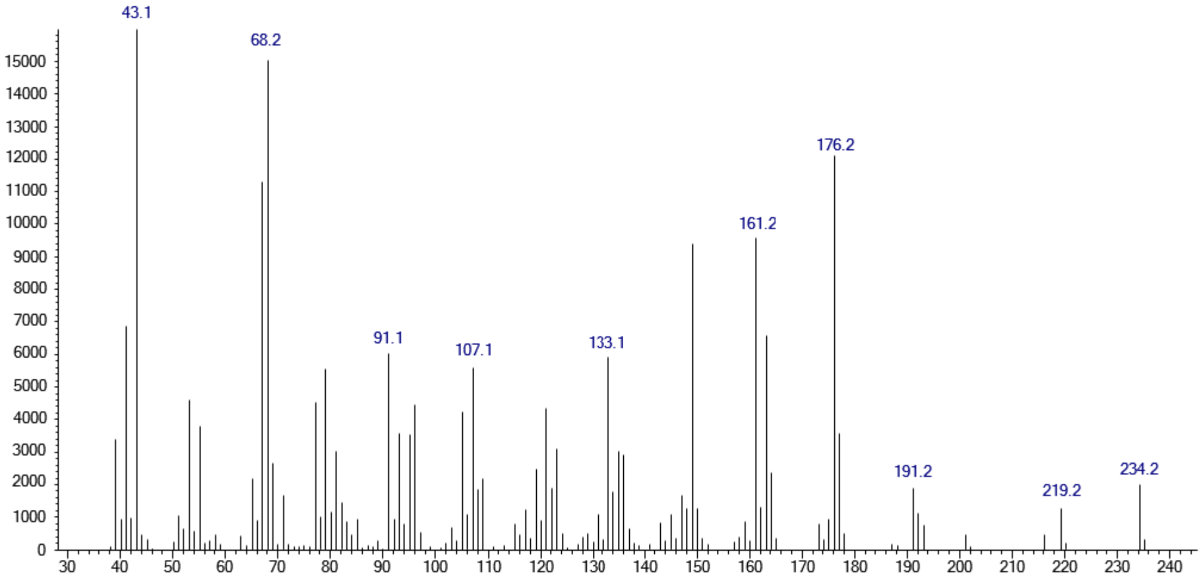

Supplementary Materials

Figure S7: Mass spectrum of Unidentified-6 detected in the essential oil of *D. botryoides* (RRI$_{Exp}$: 3103; relative content: 3.2%).

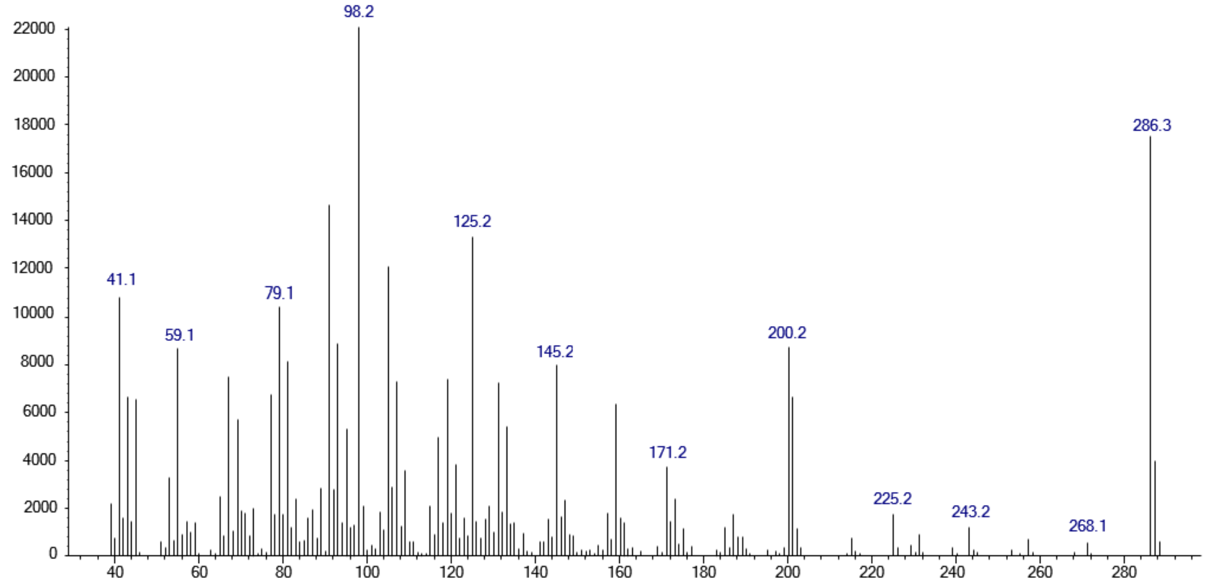

Figure S8: Mass spectrum of Unidentified-7 detected in the essential oil of *D. botryoides* (RRI$_{Exp}$: 3167; relative content: 1.0%).

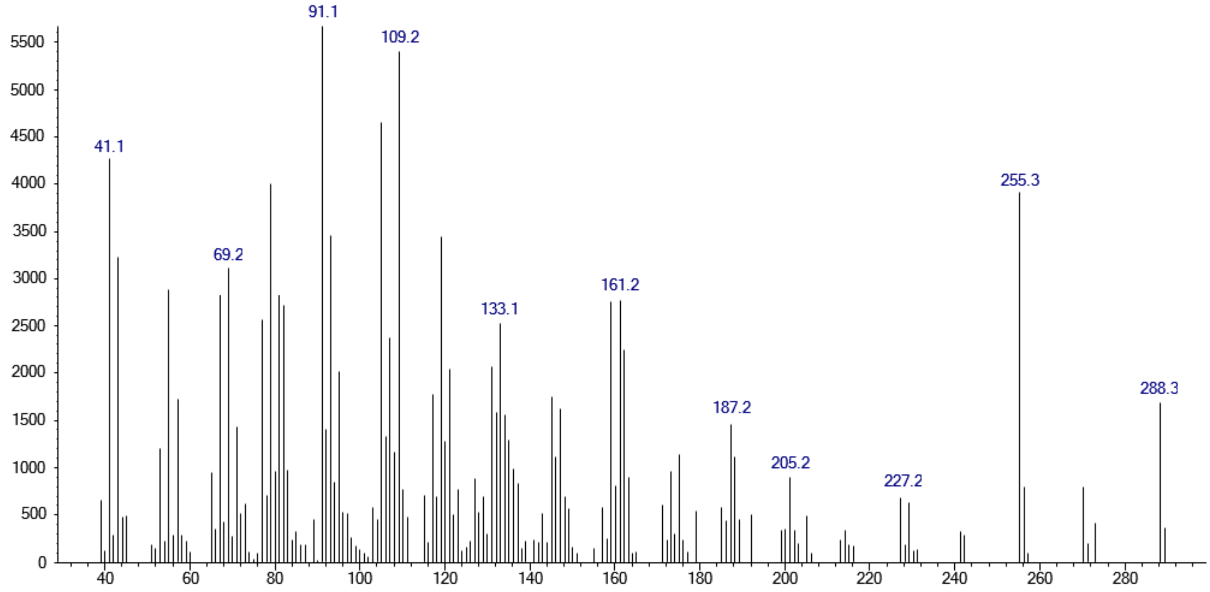

Supplementary Materials

Figure S9: Mass spectrum of Unidentified-8 detected in the essential oil of *D. botryoides* (RRI$_{Exp}$: 3230; relative content: 1.0%)

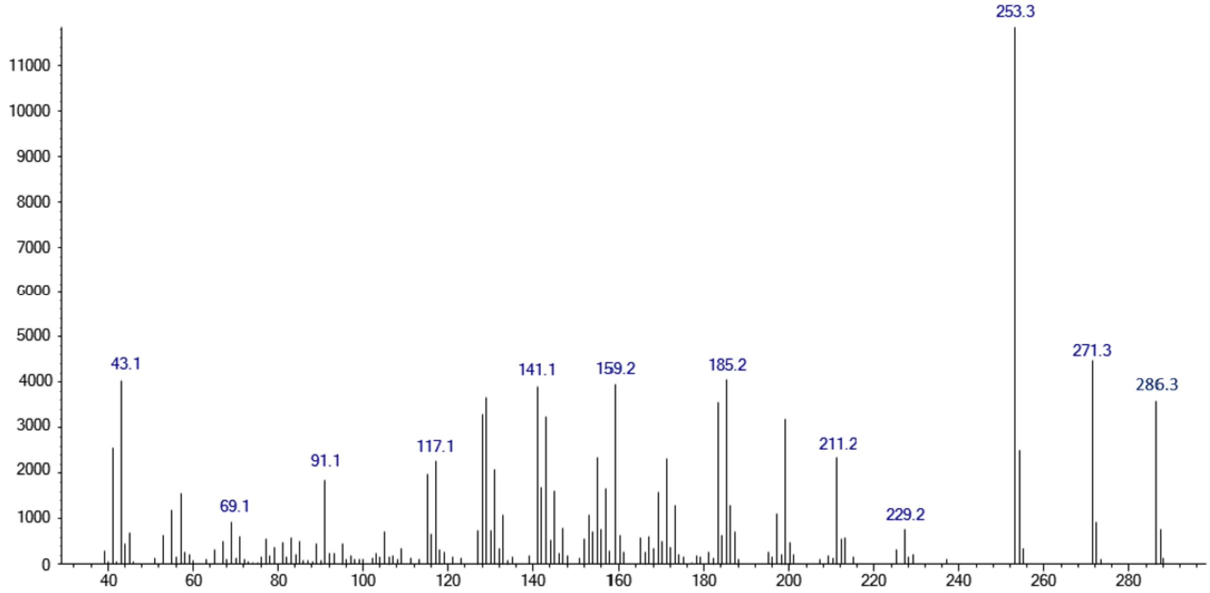

Figure S10: Mass spectrum of Unidentified-9 detected in the essential oil of *D. botryoides* (RRI$_{Exp}$: 3239; relative content: 6.0%).

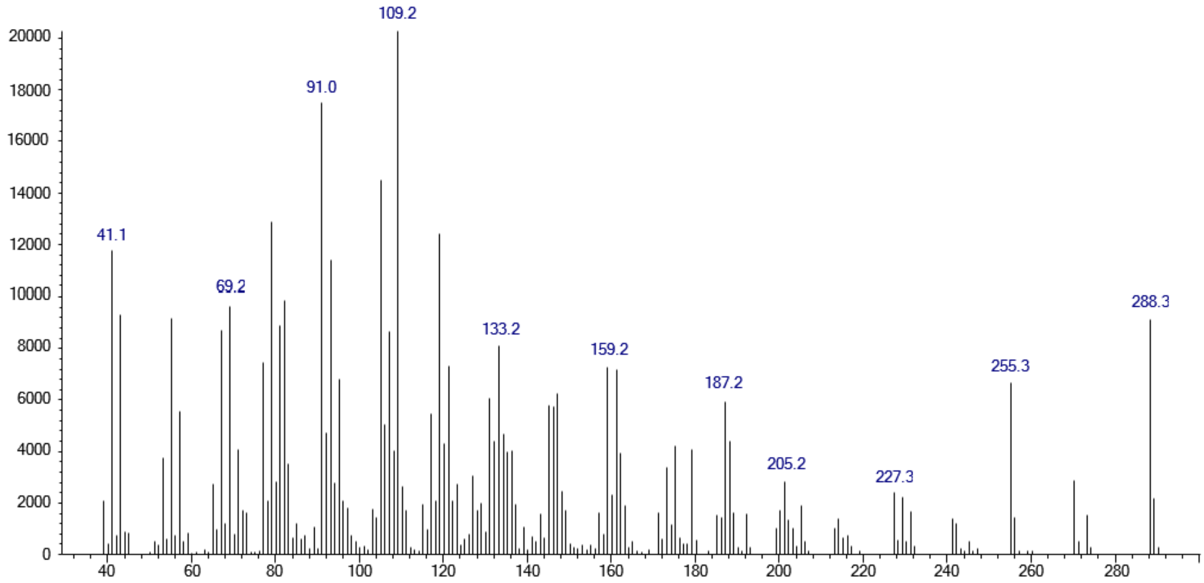

Supplement: Supplementary file 1 [file plants-15-01416-s001.zip › plants-4247421-supplementary.pdf]
